# Supplementary material for: Co-variability of the summer NDVIs on the eastern Tibetan Plateau and in the Lake Baikal region: Associated climate factors and atmospheric circulation
Source: PLoS One. 2020 Oct 28;15(10):e0239465. doi: 10.1371/journal.pone.0239465 (PMC7592756; doi:10.1371/journal.pone.0239465)
Supplement: S1 File — Highlights in the article. (DOCX) [file pone.0239465.s002.docx]

- Co-variability of NDVI between Tibetan Plateau and Lake Baikal, Siberia was found.
- An atmospheric dipole was revealed to be responsible for this co-variability.
- Optimum SSTA pattern related to the dipole and the co-variability was reported.
