# Supplementary material for: Co-variability of the summer NDVIs on the eastern Tibetan Plateau and in the Lake Baikal region: Associated climate factors and atmospheric circulation
Source: PLoS One. 2020 Oct 28;15(10):e0239465. doi: 10.1371/journal.pone.0239465 (PMC7592756; doi:10.1371/journal.pone.0239465)
Supplement: S1 Data — (ZIP) [file pone.0239465.s001.zip › Data File/Data information.docx]

**GrADS、matlab、NCL was used to process the data and all the figures was drawn by GrADS.** **All the data was uploaded to the submission system as a compressed file (Data File.zip)**

**The information of the data used for each figure and table in the manuscript is described as follows:**

**Fig 1:**

“ndvi_summer_1982_2014_720_360.nc” is the summer NDVI field during the time period 1982–2014

“PRE_summer_1982_2014.grd” is the summer precipitation field during the time period 1982–2014

**Fig 2:**

“NDVI_ETP_x1_index_summer_raw_1982_2014.grd” is the eastern Tibetan Plateau NDVI index

“NDVI_ETP_x1_index_summer_raw_1982_2014_detrend.grd” is the detrended eastern Tibetan Plateau NDVI index

“NDVI_Baikal_7_new_summer_raw_1982_2014.grd” is the Lake Baikal NDVI index

“NDVI_Baikal_7_new_summer_raw_1982_2014_detrend.grd” is the detrended Lake Baikal NDVI index

“ndvi_summer_1982_2014_720_360.nc” is the summer NDVI field during the time period 1982–2014

“ndvi_summer_1982_2014_720_360_detrended.nc” is the detrended summer NDVI field during the time period 1982–2014

**Fig 3:**

“NDVI_ETP_x1_index_summer_raw_1982_2014_std.grd” is the normalized eastern Tibetan Plateau NDVI index

“NDVI_Baikal_7_new_summer_raw_1982_2014_std.grd” is the normalized Lake Baikal NDVI index

“NDVI_ETP_x1_index_summer_raw_1982_2014_detrend_std.grd” is the normalized and detrended eastern Tibetan Plateau NDVI index

“NDVI_Baikal_7_new_summer_raw_1982_2014_detrend_std.grd” is the normalized and detrended Lake Baikal NDVI index

**Fig 4:**

“NDVI_ETP_x1_index_summer_raw_1982_2014_detrend.grd” is the detrended eastern Tibetan Plateau NDVI index

“NDVI_Baikal_7_new_summer_raw_1982_2014_detrend.grd” is the detrended Lake Baikal NDVI index

“hgt_200_summer_1982_2014_detrend.nc” is the detrended summer 200 hPa geopotential height field during the time period 1982–2014

**Fig 5:**

“hgt_200_TP_NS_pattern_summer_1982_2014_detrend_std_evf.grd” is the first and second EOF mode for the normalized summer 200 hPa geopotential height field during the time period 1982–2014

**Fig 6:**

“hgt_200_TP_NS_pattern_summer_1982_2014_detrend_std_tcf_2_std.grd” is the normalized Tibetan Plateau–Lake Baikal dipole index

“hgt_200_summer_1982_2014.nc” is the summer 200 hPa geopotential height field during the time period 1982–2014

“hgt_200_summer_1982_2014_detrend.nc” is the detrended summer 200 hPa geopotential height field during the time period 1982–2014

**Fig 7:**

“hgt_200_TP_NS_pattern_summer_1982_2014_detrend_std_tcf_2_std.grd” is the normalized Tibetan Plateau–Lake Baikal dipole index

“hgt_200_summer_1982_2014_detrend.nc” is the detrended summer 200 hPa geopotential height field during the time period 1982–2014

“hgt_600_summer_1982_2014_detrend.nc” is the detrended summer 600 hPa geopotential height field during the time period 1982–2014

“uwnd_200_summer_1982_2014_detrend.nc” is the detrended summer 200 hPa *U*-wind field during the time period 1982–2014

“uwnd_600_summer_1982_2014_detrend.nc” is the detrended summer 600 hPa *U*-wind field during the time period 1982–2014

“vwnd_200_summer_1982_2014_detrend.nc” is the detrended summer 200 hPa *V*-wind field during the time period 1982–2014

“vwnd_600_summer_1982_2014_detrend.nc” is the detrended summer 600 hPa *V*-wind field during the time period 1982–2014

**Fig 8:**

“hgt_200_TP_NS_pattern_summer_1982_2014_detrend_std_tcf_2.grd” is the Tibetan Plateau–Lake Baikal dipole index

“PRE_summer_1982_2014_detrend.nc” is the detrended summer precipitation field during the time period 1982–2014

“SAT_GHCN_summer_1982_2014_detrend.nc” is the detrended summer surface air temperature (SAT) field during the time period 1982–2014

**Fig 9:**

“SST_summer_1982_2014_detrend.nc” is the detrended summer SST field during the time period 1982–2014

“hgt_200_TP_NS_pattern_summer_1982_2014_detrend_std_tcf_2.grd” is the Tibetan Plateau–Lake Baikal dipole index

“hgt_200_TP_NS_pattern_summer_1982_2014_detrend_std_tcf_2_std.grd” is the normalized Tibetan Plateau–Lake Baikal dipole index

“SST_NWP_TEP_TA_index.grd” is the synergistic SST index

“SST_NWP_TEP_TA_index_std.grd” is the normalized synergistic SST index

**Fig 10:**

“SST_TNP_index_summer_raw_1982_2014_detrend.grd” is the tropical eastern Pacific Ocean SST index

“SST_SNA_index_summer_raw_1982_2014_detrend.grd” is the subtropical north Atlantic Ocean SST index

“SST_SWP_index_summer_raw_1982_2014_detrend.grd” is the northwest Pacific Ocean SST index

“SST_NWP_TEP_TA_index.grd” is the synergistic SST index

“hgt_200_summer_1982_2014_detrend.nc” is the detrended summer 200 hPa geopotential height field during the time period 1982–2014

**Fig 11:**

“SST_SNA_index_summer_raw_1982_2014_detrend_std_1.grd” is the negative $I_{SN-Atlantic}$

“vwnd_200_summer_1982_2014_detrend.nc” is the detrended summer 200 hPa *V*-wind field during the time period 1982–2014

**Fig 12:**

“SST_SWP_index_summer_raw_1982_2014_detrend_std.grd” is the normalized northwest Pacific Ocean SST index

“hgt_multi_summer_1982_2014_detrend.nc” is the detrended summer geopotential height field during the time period 1982–2014

“vwnd_multi_summer_1982_2014_detrend.nc” is the detrended summer *V*-wind field during the time period 1982–2014

“omega_multi_summer_1982_2014_detrend.nc” is the detrended summer omega field during the time period 1982–2014

“hgt_200_SWP_index_summer_raw_1982_2014_detrend_std.grd” is the normalized regional mean 200-hPa geopotential height to the east of Japan (32–42° N, 145–165° E)

**Table 1:**

“PRE_ETP_x1_index_summer_raw_1982_2014_detrend.grd” is the detrended eastern Tibetan Plateau precipitation index

“PRE_Baikal_7_new_summer_raw_1982_2014_detrend.grd” is the detrended Lake Baikal precipitation index

“SAT_GHCN_ETP_x1_index_summer_raw_1982_2014_detrend.grd” is the detrended eastern Tibetan Plateau surface air temperature (SAT) index

“SAT_GHCN_Baikal_7_new_summer_raw_1982_2014_detrend.grd” is the detrended Lake Baikal surface air temperature (SAT) index

“OLR_ETP_x1_index_summer_raw_1982_2014_detrend.grd” is the detrended eastern Tibetan Plateau OLR index

“OLR_Baikal_7_new_summer_raw_1982_2014_detrend.grd” is the detrended Lake Baikal OLR index

“DSWRF_ETP_x1_index_summer_raw_1982_2014_detrend.grd” is the detrended eastern Tibetan Plateau solar radiation index

“DSWRF_Baikal_7_new_summer_raw_1982_2014_detrend.grd” is the detrended Lake Baikal solar radiation index

**Discussion:**

“NDVI_ETP_x1_index_summer_raw_1982_2014_detrend.grd” is the detrended summer eastern Tibetan Plateau NDVI index

“NDVI_ETP_x1_index_spring_1982_2014_detrend.grd” is the detrended spring eastern Tibetan Plateau NDVI index

“NDVI_Baikal_7_new_summer_raw_1982_2014_detrend.grd” is the detrended summer Lake Baikal NDVI index

“NDVI_Baikal_7_new_spring_raw_1982_2014_detrend.grd” is the detrended spring Lake Baikal NDVI index

**The information of the data used for each figure in the Response to Reviewer 1 is described as follows:**

**Fig R. 1:**

“ndvi_summer_1982_2014_720_360_01.bin” is the summer NDVI field during the time period 1982–2014, in which the NDVI values higher than 0.1 are removed.

**Fig R. 2:**

“ndvi_summer_1982_2014_720_360_01.bin” is the summer NDVI field during the time period 1982–2014, in which the NDVI values higher than 0.1 are removed.

**Fig R. 3:**

“NDVI_Baikal_7_new_summer_raw_1982_2014_detrend.grd” is the detrended Lake Baikal NDVI index

“SHTFL_summer_1982_2014_detrend.nc” is the detrended summer sensible heat net flux field during the time period 1982–2014

“gflux_summer_1982_2014_detrend.nc” is the detrended summer surface heat flux field during the time period 1982–2014

**Fig R. 4:**

“NDVI_ETP_x1_index_summer_raw_1982_2014_detrend.grd” is the detrended eastern Tibetan Plateau NDVI index

“SHTFL_summer_1982_2014_detrend.nc” is the detrended summer sensible heat net flux field during the time period 1982–2014

“gflux_summer_1982_2014_detrend.nc” is the detrended summer surface heat flux field during the time period 1982–2014

**Fig R. 5:**

“NDVI_ETP_x1_index_summer_raw_1982_2014_detrend_std.grd” is the normalized and detrended eastern Tibetan Plateau NDVI index

“NDVI_Baikal_7_new_summer_raw_1982_2014_detrend_std.grd” is the normalized and detrended Lake Baikal NDVI index

“hgt_multi_summer_1982_2014_detrend.nc” is the detrended summer geopotential height field during the time period 1982–2014

“vwnd_multi_summer_1982_2014_detrend.nc” is the detrended summer *V*-wind field during the time period 1982–2014

“omega_multi_summer_1982_2014_detrend.nc” is the detrended summer omega field during the time period 1982–2014

**Fig R. 6:**

“SST_NWP_TEP_TA_index.grd” is the synergistic SST index

“SST_summer_1982_2014_detrend.nc” is the detrended summer SST field during the time period 1982–2014

**Fig R. 7:**

“SST_joint_index_summer_1982_2014.dat” is the summer joint index of $\text{I}_{\text{NIO}}$ and $\text{I}_{\text{TE-Pacific}}$

“hgt_200_summer_1982_2014_detrend.nc” is the detrended summer 200 hPa geopotential height field during the time period 1982–2014

**The information of the data used for each figure in the Response to Reviewer 2 is described as follows:**

**Fig R. 1:**

“ndvi_summer_1982_2014_720_360.nc” is the summer NDVI field during the time period 1982–2014

**Fig R. 2:**

“hgt_200_TP_NS_pattern_summer_1982_2014_detrend_std_tcf_2_std.grd” is the normalized Tibetan Plateau–Lake Baikal dipole index

“SST_NWP_TEP_TA_index_std.grd” is the normalized synergistic SST index

**Fig R. 3:**

“SST_TNP_index_summer_raw_1982_2014_detrend.grd” is the tropical eastern Pacific Ocean SST index

“SST_SNA_index_summer_raw_1982_2014_detrend.grd” is the subtropical north Atlantic Ocean SST index

“SST_SWP_index_summer_raw_1982_2014_detrend.grd” is the northwest Pacific Ocean SST index

“SST_NWP_TEP_TA_index.grd” is the synergistic SST index

“hgt_200_summer_1982_2014_detrend.nc” is the detrended summer 200 hPa geopotential height field during the time period 1982–2014

**Fig R. 4:**

“SST_NWP_TEP_TA_index.grd” is the synergistic SST index

“SST_summer_1982_2014_detrend.nc” is the detrended summer SST field during the time period 1982–2014

**Fig R. 5:**

“SST_SNA_index_summer_raw_1982_2014_detrend_std_1.grd” is the negative $I_{SN-Atlantic}$

“vwnd_200_summer_1982_2014_detrend.nc” is the detrended summer 200 hPa *V*-wind field during the time period 1982–2014

**Fig R. 6:**

“SST_SWP_index_summer_raw_1982_2014_detrend_std.grd” is the normalized northwest Pacific Ocean SST index

“hgt_multi_summer_1982_2014_detrend.nc” is the detrended summer geopotential height field during the time period 1982–2014

“vwnd_multi_summer_1982_2014_detrend.nc” is the detrended summer *V*-wind field during the time period 1982–2014

“omega_multi_summer_1982_2014_detrend.nc” is the detrended summer omega field during the time period 1982–2014

“hgt_200_SWP_index_summer_raw_1982_2014_detrend_std.grd” is the normalized regional mean 200-hPa geopotential height to the east of Japan (32–42° N, 145–165° E)
