# Supplementary material for: Co-variability of the summer NDVIs on the eastern Tibetan Plateau and in the Lake Baikal region: Associated climate factors and atmospheric circulation
Source: PLoS One. 2020 Oct 28;15(10):e0239465. doi: 10.1371/journal.pone.0239465 (PMC7592756; doi:10.1371/journal.pone.0239465)
Supplement: Attachment — Submitted filename: PONE-D-20-11620-Response_to_Reviewer 2.docx [file pone.0239465.s003.docx]

***Response to Reviewer 2***

We appreciate the detailed comments and suggestions from the reviewer, which were very valuable for improving the manuscript. In particular, the reviewer expressed concern about the physical link between the Tibetan Plateau–Lake Baikal dipole pattern and the SSTAs in different oceans, which was very important and motivated us to further explore it in the revised manuscript. We have considered these comments seriously and revised the manuscript accordingly. Our responses to the comments are summarized as follows. The associated revisions in the manuscript are highlighted in red.

1) *In the Introduction and Discussion as well as Conclusion, the scientific implications and novelty of this study have not been clarified. In the revision, it is suggested to add more scientific implications on the climatology and ecology.*

**Reply:** Thanks for your suggestion. In this paper, we found a co-variability of the summer NDVIs between the Tibetan Plateau and Siberia and revealed the potential reason for this co-variability. This is a novelty that has never been reported before. To emphasize this new finding and its scientific implications, we have added a paragraph in the revised manuscript as follows.

“The characteristics of the NDVI over the Tibetan Plateau and Siberia and their corresponding climate factors have been widely studied, respectively (Suzuki et al., 2000; Ding et al., 2007; Park et al., 2010; Mao et al., 2012; Sun et al., 2016; Hua et al., 2018). To our knowledge, there has no study on the relationship between the NDVI in the two regions. The co-variability of the summer NDVIs in the two regions is a new finding. Investigating climate factors and atmospheric circulation related to this co-variability may improve the understanding of the response of regional vegetation to climate change and clarify the reason for the synergistic change of vegetation in different regions. This study is possibly conducive to recognizing the contribution of atmospheric circulation and associated climate factors to the terrestrial ecosystem from a new aspect (i.e., the co-variability of vegetation), and therefore may provide a scientific basis for the organization and cooperation of ecological and environmental protection among countries and areas.” In addition, we have also added several sentences to state scientific implications in the last paragraph of this paper. See lines 88–99 on pp. 4–5 and lines 696–703 on pp. 32–33.

2) *L79-82 presents that NDVI variations over the two regions were characterized by uptrend. However, Figure 3 shows that there is no an increasing trend. How to explain the paradoxes?*

**Reply:** This paradox may be owing to the spatial heterogeneity of the response of the NDVI to climate change. The vegetation types gradually change from forest and shrub over the southeastern Tibetan Plateau to meadow, grass, and desert vegetation over the northwestern Tibetan Plateau (Pang et al., 2016). This diversity of vegetation types causes various responses of the NDVI to climate change. Previous studies mainly focused on the NDVI over almost the whole Tibetan Plateau in growing season, which showed an increasing trend (Pang et al., 2016; Du et al., 2016). Our study focused on the NDVI over a smaller region (i.e., the eastern Tibetan Plateau). The NDVI over this region showed a decreasing trend although the NDVI over most of the Tibetan Plateau showed increasing trends (Fig R. 1).

As shown in Fig R. 1, the increasing and decreasing trends in NDVI are scattered over the Lake Baikal region. The area-mean NDVI over the Lake Baikal region shows a weak increasing trend of 0.11/decade. This trend is generally consistent with previous studies (Gong et al., 2002), although the specific values of trends are different because of selecting different regions in Siberia.

We expected that the NDVI over the Tibetan Plateau is linked with that over Siberia due to the similar decadal trend under the background of global warming. But we found this co-variability between the two regions is very clear on an interannual timescale. This is an interesting finding and is also an important reason for comparing the two regions.

In the revised manuscript, we have briefly explained the difference of the NDVI trend between different parts of the eastern Tibetan Plateau. This paragraph has been rewritten as follows. “The NDVI over the Lake Baikal region shows a weak increasing trend of 0.11/decade, which is generally consistent with previous studies (Gong et al., 2002), although the specific values of trends are different because of selecting different regions in Siberia. The NDVI over the eastern Tibetan Plateau shows a decreasing trend of –0.33/decade, whereas the NDVI over the central and western Tibetan Plateau generally shows an increasing trend (not shown). This difference may be attributable to different vegetation types, leading to the spatial heterogeneity of the response of the NDVI to climate change over the Tibetan Plateau (Zhang et al., 2013; Pang et al., 2016).” See lines 261–268 on pp. 12–13.

**Fig R. 1** Spatial distribution of the normalized summer NDVI trend during the time period 1982–2014. The black boxes from top to bottom represent the Lake Baikal and the eastern Tibetan Plateau regions, respectively.

3) *Please clarify the data source of solar radiation in the Table 1.*

**Reply:** The downward shortwave radiation flux on a resolution of 192×94 Gaussian grid was obtained from the NCEP/NCAR Reanalysis dataset, which reflects the solar radiation received by the Earth’s surface. This data information has been added in the revision (see lines 143–145 on p. 7).

4) *L484. Please clarify the physical implication of this SST index. This study did not explore the possible physical links between SST anomaly and TP-LB dipole index. Is there possibly no physical link? Are they separated and derived from atmospheric oscillation and ocean oscillation, respectively?*

**Reply:** In the revised manuscript, we constructed an empirical statistical model to fit the Tibetan Plateau-Lake Baikal dipole index using the method of stepwise regression, which can effectively select optimal factors that are independent of each other. The model was established as follows:

$I_{Dipole}={0.429I}_{NW-Pacific}-{0.367I}_{SN-Atlantic}-0.283I_{TE-Pacific}$,

in which $I_{Dipole}$ is the Tibetan Plateau-Lake Baikal dipole, and $I_{NW-Pacific}$,$I_{SN-Atlantic}$, and $I_{TE-Pacific}$ represent the SSTs regionally averaged over the northwest Pacific Ocean, the subtropical North Atlantic Ocean, and the tropical eastern Pacific Ocean, respectively. The northern Indian Ocean SST index ($I_{NIO}$), which is defined as the SSTs regionally over the northern Indian Ocean (5–25° N, 50–70° E), was removed from this model after stepwise regression processes. All these indices were normalized. The right terms can reflect the effects of the synergistic SSTAs in these oceans with different weights and therefore are referred to as the synergistic SST index. Fig R. 2 shows that the variability of the synergistic SST index is in good agreement with that of the Tibetan Plateau-Lake Baikal dipole index, with a high correlation of 0.72 (significant at the 99.9% confidence level). In other words, this synergistic SST index in the revised manuscript can reflect the variability of the Tibetan Plateau-Lake Baikal dipole better than the original one.

The correlation coefficient between the synergistic SST index and the Tibetan Plateau/Lake Baikal NDVI index is 0.45/0.40 (both significant at the 95% confidence level). These results imply that this configuration of SSTAs seems to regulate the Tibetan Plateau–Lake Baikal dipole pattern of atmospheric circulation and subsequently causes the co-variability of the NDVI between the eastern Tibetan Plateau and Lake Baikal regions.

**Fig R. 2** Time series of the normalized Tibetan Plateau–Lake Baikal dipole (black line) and synergistic SST (red line) indices during the time period 1982–2014. TP–LB, Tibetan Plateau–Lake Baikal.

Next, we explore the potential contributions of SSTAs in different regions to the variability of the Tibetan Plateau–Lake Baikal dipole, respectively. Cooler (warmer) SSTs in the tropical eastern Pacific correspond to anomalous downward (upward) motion in the tropical eastern Pacific and anomalous upward (downward) motion around the western North Pacific through enforcing (restraining) the Pacific Walker circulation (Deser and Wallace, 1990; Trenberth and Caron, 2000; Yan et al., 2011). As a result of the above process, negative (positive) geopotential height anomalies appear around southern China and the South China Sea in correspondence to negative (positive) SSTAs in the tropical eastern Pacific. This can be detected in the correlation between the summer negative $I_{TE-Pacific}$ and the simultaneous 200 hPa geopotential heights (Fig R. 3a). For ease of understanding, we used the negative $I_{TE-Pacific}$ to perform correlation analysis since the negative $I_{TE-Pacific}$ corresponds to the positive Tibetan Plateau–Lake Baikal dipole index. As shown in Fig R. 3a, the SSTAs in the tropical eastern Pacific may primarily modulate the south pole of the Tibetan Plateau-Lake Baikal dipole.

**Fig R. 3** Distribution of the correlation coefficients between the summer (a) negative tropical eastern Pacific Ocean SST index/(b) negative subtropical north Atlantic Ocean SST index/(c) positive northwest Pacific Ocean SST index/(d) synergistic SST index and the simultaneous 200 hPa geopotential height field during the time period 1982–2014. The yellow (red) shading denotes a positive correlation significant at the 95% (99%) confidence level and the blue (purple) shading denotes a negative correlation significant at the 95% (99%) confidence level.

Note that the correlation between the summer synergistic SST index and the simultaneous SST field (Fig R. 4) shows that apart from significant correlations over the northwest Pacific Ocean, the subtropical North Atlantic Ocean, and the tropical eastern Pacific Ocean, a significant negative correlation appears over the northern Indian Ocean. The $I_{NIO}$ is highly correlated with the synergistic SST index, with a correlation coefficient of −0.61 (significant at the 99.9% confidence level). This result implies that the synergistic SST index also reflects the variability of the $I_{NIO}$ though the synergistic SST domain does not cover the northern Indian Ocean. The correlation between the summer negative $I_{NIO}$ and the simultaneous 200 hPa geopotential heights (figure omitted) reveals that negative correlations appear around southern China and the South China Sea, resembling Fig R. 3a. This suggests that the variability of the SSTs in the northern Indian Ocean may also play an important role in modulating the south part of the Tibetan Plateau–Lake Baikal dipole. The SSTAs in the tropical eastern Pacific and the northern Indian Ocean seems to affect the south part of this dipole through a pair of gears (strong coupling) between the Indo-monsoon circulation and the Pacific Walker circulation (GIP; Wu and Meng, 1998; Meng and Wu, 2000).

**Fig R. 4** Distribution of the correlation coefficients between the summer synergistic SST index and the simultaneous SST field during the time period 1982–2014. The yellow (red) shading denotes a positive correlation significant at the 95% (99%) confidence level and the blue (purple) shading denotes a negative correlation significant at the 95% (99%) confidence level.

The SSTAs in the tropical Atlantic can affect downstream climate through stimulating wave train pattern (Wu et al., 2001; Zuo et al., 2013; Wang et al., 2017). The correlation between the negative $I_{SN-Atlantic}$ between 200 hPa *V*-winds (Fig R. 5) shows two wave trains from the Atlantic to East Asia at the mid-low and mid-high latitudes, respectively, in which the wave train at the mid-low latitudes is generally similar to the Silk Road teleconnection (Lu et al., 2002; Hong and Lu, 2016). Through exciting the wave trains, the SSTAs in the subtropical North Atlantic Ocean result in geopotential height anomalies around the Lake Baikal (Fig R. 3b)—that is, the $I_{SN-Atlantic}$ may modulate the north part of the Tibetan Plateau–Lake Baikal dipole.

**Fig R. 5** Distribution of the correlation coefficients between the summer negative subtropical north Atlantic Ocean SST index and the simultaneous 200 hPa *V*-wind field during the time period 1982–2014. The yellow (red) shading denotes a positive correlation significant at the 90% (95%) confidence level and the blue (purple) shading denotes a negative correlation significant at the 90% (95%) confidence level. The black dashed lines denote the Rossby wave trains.

The vertical circulation and geopotential height anomalies regressed upon the $I_{NW-Pacific}$ on a cross section along 155 °E show anomalous upward motions over the northwest Pacific Ocean and correspondingly a significant positive anomaly appears in the upper troposphere around 40° N, to the east of Japan (Fig R. 6a), which can be regarded as a result of a warmer SST forcing. The 200-hPa geopotential height anomalies regressed upon the area-mean 200-hPa geopotential height to the east of Japan (32–42° N, 145–165° E) shows a significant positive anomaly around the Lake Baikal (Fig R. 6b), implies that when a blocking-like high-pressure anomaly appears to the east of Japan, the high-pressure anomalies (i.e., the northeast Asian blocking high) tend to occur around the Lake Baikal. Through forcing the overlying atmospheric circulation anomalies over the northwestern Pacific, the $I_{NW-Pacific}$ seems to modulate the north part of the Tibetan Plateau–Lake Baikal dipole. Therefore, the $I_{NW-Pacific}$ is significantly correlated with geopotential potential heights around the Lake Baikal (Fig R. 3c).

**Fig R. 6** (a) Summer vertical motion and geopotential height anomalies regressed upon the northwest Pacific Ocean SST index on a cross section along 155 °E. (b) The 200 hPa geopotential height anomalies regressed on the area-mean 200-hPa geopotential height to the east of Japan (32–42° N, 145–165° E) during the time period 1982–2014. The yellow (red) shading denotes a positive correlation significant at the 95% (99%) confidence level and the blue (purple) shading denotes a negative correlation significant at the 95% (99%) confidence level.

In summary, the synergistic SST index effectively reflects a pattern of the SSTAs in the tropical eastern Pacific Ocean, the northern Indian Ocean, the subtropical North Atlantic Ocean, and the northwest Pacific Ocean (Fig R. 4). Through the aforementioned physical processes, the SSTAs in different regions seem to affect different parts of the Tibetan Plateau–Lake Baikal dipole and jointly modulate this dipole pattern. Thus, the synergistic SSTA pattern is closely linked with this dipole pattern (Fig R. 3d).

The aforementioned results imply a potential physical link between the SSTA pattern and the Tibetan Plateau–Lake Baikal dipole. However, the physical mechanism requires further investigation in detail. For example, we need to further explore why a blocking-like high-pressure anomaly to the east of Japan, which is forced by the SSTAs in the northwest Pacific, is conducive to the high-pressure anomalies around the Lake Baikal (the northeast Asian blocking high).

Thank you for asking the question, which reminds us of what the potential readers are interested in. To answer this question, we have preliminarily added the possible physical links as mentioned above. See lines 506–615 on pp. 24–29 and Figures 9–12 in the revised manuscript. The references related to this discussion have also been listed in the revised manuscript.
