# Supplementary material for: Co-variability of the summer NDVIs on the eastern Tibetan Plateau and in the Lake Baikal region: Associated climate factors and atmospheric circulation
Source: PLoS One. 2020 Oct 28;15(10):e0239465. doi: 10.1371/journal.pone.0239465 (PMC7592756; doi:10.1371/journal.pone.0239465)
Supplement: Attachment — Submitted filename: PONE-D-20-11620-R2-Response to Reviewer 1.docx [file pone.0239465.s004.docx]

***Response to Reviewer 1***

We appreciate the detailed comments and suggestions from the reviewer, which were very valuable for improving the manuscript. We have considered these comments seriously and revised the manuscript accordingly. Our responses to the comments are summarized as follows. The associated revisions in the manuscript are highlighted in red.

1) *Line51, “…vulnerable to global climate change” would be better.*

**Reply:** This sentence has been revised. See line 51 on p. 3 in the revised manuscript.

2) *Line 262,263, statistical significance for the trend is needed.*

**Reply:** Thanks for your suggestion. The NDVI over the Lake Baikal region shows a weak increasing trend of 0.11/decade, non-significant. The NDVI over the eastern Tibetan Plateau shows a decreasing trend of –0.33/decade, significant at the 90% confidence level. We have added the statistical significances in the revised manuscript. See lines 263 and 266 on p. 13.

3) *Line 286, should be play important parts in.*

**Reply:** This phrase has been corrected. See line 288 on p. 14.

4) *Line 317, this can be removed.*

**Reply:** According to this suggestion, the sentence has been removed.

5) *Line 315-321, the author stated that precipitation corresponds more clouds and less solar radiation. However, there are calculated negative correlation between NDVI and precipitation, positive correlations between NDVI and OLR, and between NDVI and solar radiation. Why* *precipitation has different sign of correlation with that of OLR?*

**Reply:** The outgoing longwave radiation (OLR) is determined by temperatures at the top of cloud. Stronger (weaker) convection, associated with higher (lower) precipitation, can lead to more (less) cloud and drive the top of cloud to a higher (lower) level. When the top of cloud is higher (lower), lower (higher) temperatures appear at its top, leading to lower (higher) OLR. As such, higher (lower) precipitation/stronger (weaker) convection corresponds to lower (higher) OLR and solar radiation. Several previous studies (Kripalani et al., 1991; Chaudhari et al., 2018) have explained the precipitation-OLR anticorrelation. It is reasonable that precipitation has different sign of correlation with that of OLR. In the revised manuscript, we have briefly presented the OLR-convection/cloud relationship in Materials (see lines 142–143 on p. 7). In addition, we found a typo in the original manuscript, which may mislead the understanding of the reviewer. This mistake has been corrected in the revised manuscript (See line 323 on p. 16).

6) *Line 341, why 200 hPa geopotential height instead of 500 hPa or other height is chosen?*

**Reply:** We calculated the correlation between the Tibetan Plateau/Lake Baikal NDVI index and geopotential height field at different levels. The north-south dipole pattern related to the NDVIs is more significant and clearer at the 200 hPa level than that at the other levels. Also, the second EOF mode of geopotential heights displays a clearer north-south dipole pattern at the 200 hPa level. Therefore, we chose 200 hPa geopotential heights to reflect the typical atmospheric circulation pattern responsible for the co-variability of the NDVIs. Furthermore, Figure 7 indicates that the out-of-phase dipole pattern appears in both the upper and mid- to lower troposphere, forming a deep configuration of high-/low-pressure anomalies. This further suggests that the 200 hPa level is suitable for our study since it can also measure the atmospheric circulation anomalies at the lower level.

7) *Figure 6, time coefficients for EOF1 and EOF2 should be given accordingly.*

**Reply:** Thanks for this suggestion. The time coefficients for EOF1 and EOF2 (i.e., PC1 and PC2) have been added in the revision as Figures 5c and 5d. The figure captions for Figures 5c and 5d have added in the revised manuscript accordingly (See lines 380–381 on p. 18 and lines 1055–1056 on p. 47).

8) *Line 400, confused about the sentence: superimposition of the geopotential height anomalies regressed on the dipole index. Is the PC2 time coefficients regression on the eddy geopotential height field? Explanation is needed.*

**Reply:** Firstly, we calculated the 200 hPa geopotential height anomalies regressed upon the dipole index (i.e., PC2). Secondly, the geopotential height anomalies were superimposed on the climatological mean 200 hPa eddy geopotential height field. Through this superimposition, we can intuitively see anomalous characteristics of the atmospheric circulation systems (i.e., the South Asian high and the northeastern Asian blocking high) associated with this dipole. In the revised manuscript, we have presented the aforementioned explanation. See lines 402–406 on p. 19.

9) *Except the climate factors, whether any anthropogenic activities have an influence on the changes in NDVI?*

**Reply:** We cannot exclude the influence of anthropogenic activities on the global and local climate and relevant changes in NDVI. However, we have reasons to believe that anthropogenic activities play an inessential role in modulating the variability of the NDVIs in the Tibetan Plateau and Lake Baikal regions.

Firstly, the Tibetan Plateau and Lake Baikal regions are both sparsely populated areas. The vegetation in the Tibetan Plateau is relatively undisturbed by anthropogenic activities due to the low population (Zhang et al., 2013; Huang et al., 2016). Also, the population density in the Lake Baikal region is low (Zemskaya et al., 2020). This suggests that the changes in vegetation over the two regions may be dominantly affected by climate factors rather than anthropogenic activities. Secondly, the probability that the co-variability between the NDVI over the eastern Tibetan Plateau and that over the Lake Baikal region is triggered by simultaneous anthropogenic activities in the two regions is very low, especially on interannual timescales.

Due to the abovementioned reasons, the influence of anthropogenic activities should not be considered as the main factor. So, we did not discuss the effect of anthropogenic activities in this paper.

**References**

Chaudhari HS, Hazra A, Pokhrel S, Chakrabarty C, Saha SK, [Pentakota](https://www.researchgate.net/profile/Sreenivas_Pentakota) S. SST and OLR relationship during Indian summer monsoon: a coupled climate modelling perspective. Meteorol. Atmos. Phys. 2018; 130: 211–225. https://doi.org/10.1007/s00703-017-0514-0.

Huang K, Zhang YJ, Zhu JT, Liu YJ, Zu JX, Zhang J. The Influences of Climate Change and Human Activities on Vegetation Dynamics in the Qinghai-Tibet Plateau. Remote Sens. 2016; 8(10): 876–893. https://doi.org/10.3390/rs8100876.

Kripalani RH, Singh SV, Arkin PA. Large scale features of rainfall and outgoing long wave radiation over India and adjoining regions. Contrib. Atmos. Phys. 1991; 64: 159–168.

Zemskaya TI, Cabello-Yeves PJ, Pavlova ON, Rodriguez-Valera F. Microorganisms of Lake Baikal—the deepest and most ancient lake on Earth. Appl. Microbiol. Biotechnol. 2020; 104: 6079–6090. https://doi.org/10.1007/s00253-020-10660-6.

Zhang L, Guo HD, Ji L, Lei LP, Wang CZ, Yan DM. et al. Vegetation greenness trend (2000 to 2009) and the climate controls in the Qinghai-Tibetan Plateau. J. Appl. Remote Sens. 2013; 7(1): 073572. https://doi.org/10.1117/1.JRS.7.073572.
